# Predictors of student preparedness for advanced pharmacy practice experiences

**DOI:** 10.1186/s12909-024-05287-4

**Published:** 2024-03-16

**Authors:** Shantanu Rao

**Affiliations:** College of Pharmacy, The University of Findlay, 1000 N. Main Street, 45840 Findlay, OH USA

**Keywords:** Top drug competency, APPE readiness, Capstone, PCOA

## Abstract

**Background:**

A capstone course often serves as the final checkpoint of student readiness before the commencement of experiential training. The purpose of this study was to determine if the assessment components from the capstone course can serve as predictors of student performance during their Advanced Pharmacy Practice Experiences (APPEs).

**Methods:**

Student data was analyzed to observe the correlation between performance in the Pharmacy Curriculum Outcomes Assessment (PCOA), student performance in the capstone course, and the overall grade point average (GPA) earned during APPEs. Spearman rank correlation analysis, multiple linear regression, and Mann-Whitney U test were used for statistical analysis.

**Results:**

A statistically significant positive correlation was observed between the overall APPE GPA and students’ capstone course grade, top drug competency exam score, pharmacy calculation competency exam score, and PCOA exam score. A significant regression equation was obtained during the analysis: (F(5, 97) = 5.62, *p* < 0.001), with an R2 = 0.225 (adjusted R2 = 0.185). In the linear regression model, capstone GPA emerged as a significant predictor (β = 0.155; *p* = 0.019) of APPE GPA amongst the tested variables. Additionally, students scoring < 73% on the top drug competency exam in the capstone course or less than the reference group in the PCOA exam were found to have significantly lower GPA during their APPEs compared to other students.

**Conclusion:**

Performance on the top drug competency exam and the PCOA exam can serve as potential predictors of success during APPEs.

## Background

Advanced pharmacy practice experiences (APPEs) are an integral part of the pharmacy curriculum and provide pharmacy students with a wide-ranging, valuable clinical experience of patient care under the guidance of pharmacist preceptors [1]. Accordingly, the Accreditation Council of Pharmacy Education (ACPE) encourages Doctor of Pharmacy (Pharm.D.) degree-offering institutions to ascertain student proficiency in pre-APPE pharmacy curriculum ‘*to ensure student readiness to enter APPE*’ [1]. Intuitively, recent studies have examined the possibility of determining APPE readiness amongst pharmacy students. The results from these studies have identified several possible indicators of APPE readiness including the Pharmacy Curriculum Outcomes Assessment (PCOA) score [2], student performance on classroom-based patient case activity [3], poor academic performance and professionalism issues [4], student understanding regarding the patient workup process [5], and workplace participation [6].

Over the years, the inclusion of a capstone course has been successfully utilized by pharmacy programs to both assess and enhance clinical problem-solving skills among pharmacy students [7–9]. To maintain consistency regarding patient care across the various pharmacy settings, the Joint Commission of Pharmacy Practitioners (JCPP) has outlined the Pharmacist’s Patient Care Process (PPCP) [10]. Implementation of the PPCP was subsequently demarcated in the ACPE 2016 standards [1] and a capstone course is frequently utilized to assess students’ ability to apply components of the PPCP [8, 11, 12]. Capstone courses often include a variety of strategies for the determination of clinical assessment and communication skills in students such as patient case-based discussions, student presentations, and Objective Structured Clinical Examinations (OSCEs) [8, 13, 14]. Therefore, performance in these assessments within a capstone course may serve as an indicator of practice readiness amongst pharmacy students.

Capstone course is an integral part of the pharmacy curriculum at our institution as well and includes all of the above-mentioned assessment approaches to evaluate clinical skills amongst pharmacy students. Since this course is offered over the spring semester of the third professional year, the assessment components within the capstone course have the potential to serve as a measure of student readiness for their upcoming APPE rotations. Attempting the PCOA exam is required of students during the spring semester of their third professional year. Additionally, in the past 2 years, top drug competency and pharmacy calculations competency exams have been included within our capstone course to both promote and gauge student preparedness for their APPEs. The primary hypothesis of this data analysis was that the performance on the competency-based assessments within the capstone course will have a direct correlation with student performance during their APPEs. The objective of this retrospective analysis, therefore, was to determine if the various assessment components of the capstone course can serve as potential predictors of student performance during their APPEs.

## Method

### Capstone course overview

To identify potential predictors of student success during APPEs, student data from the capstone course of the Pharm.D. curriculum offered at the University of Findlay was analyzed in this retrospective study (*n* = 105). Capstone course data from two consecutive years of our program (class of 2021 and 2022), with comparable assessment and competency requirements in the course, was included as part of the present data analysis. Additionally, a top drug competency exam was offered, for the first time, as part of the capstone course starting in spring 2020. Given the novelty of this assessment, the present data analysis focused only on the cohorts of students who were required to demonstrate competency regarding top drug information, OSCEs, and pharmacy calculations as part of the capstone course at our institution.

Student scores on major assessments included in the capstone course were analyzed to predict student success during their APPEs. OSCEs contributed to 30% of the capstone course grade while various competency exams (∼ 5) comprised another 30% of the course grade. Discussions, case presentations, journal club, course assignments, etc. collectively were assigned 35% of the course grade. Since passing the capstone course and the individual components of the capstone course (top drug, pharmacy calculations, OSCEs) are a progression requirement for third-professional year students to start their APPE rotations, these parameters were individually analyzed as potential indicators for APPE preparedness in the present data analysis. While attempting the PCOA exam is a requirement, student preparation for the PCOA exam was encouraged by assigning 5% of the overall capstone course grade to the PCOA exam scores. For the present analysis, the PCOA exam score was considered an independent indicator of student performance during APPEs. While the spring 2020 capstone course was interrupted by the onset of the COVID-19 pandemic, the planned assessments for the course were successfully migrated to an online platform and did not significantly impact the overall course objectives.

### Capstone course data, APPE grades, and data analysis

The approach adopted by our instructors to design the top drug competency assessments of the capstone course was reported recently [15]. Briefly, students were provided a list of top drugs to review various aspects of drug information (generic/brand name, adverse effects, indication, therapeutic class, etc.) at the start of the semester, and their knowledge regarding top drug information was tested, at the end of the semester, through the top drug competency examination. Similarly, students were provided a review lecture on major pharmacy calculations, and, a few weeks later, their knowledge regarding pharmacy calculations was tested through the pharmacy calculations competency examination. Exam data from only the initial attempt of the competency exams (excluding remediation score) was considered for the present data analysis to accurately reflect the correlation between student performance in the competency exams and their preparedness for APPEs. To assess students’ clinical and communication skills, as part of the capstone course, students were assessed on up to 4 OSCEs. An overview of the logistics for the activity along with the counseling resource guide and rubric were available to students before their scheduled OSCE. The average student performance on all OSCEs (on a % scale) was used for subsequent data analysis. Finally, the PCOA reference group score, provided as part of the school’s PCOA report, was used as a cutoff to categorize the APPE grade point average (GPA) in this study. Only 2 students were unable to attempt the PCOA exam for the analyzed cohorts in this study (*n* = 103).

For the present study, the preceptor assigned grades (mean GPA) to students over their nine APPE rotations (required and elective) served as an indicator of student success in APPEs. The preceptor assigned letter grade for an APPE rotation is based upon their assessment of student performance in domains such as professionalism, communication, drug information, patient care, disease knowledge, and administrative skills. Student data from the capstone course and APPE rotations was compiled using a Microsoft (Redmond, WA) Excel spreadsheet. The correlation between the capstone course grade, competency exam scores, PCOA score, and student GPA during APPEs was assessed using the Spearman rank correlation analysis. Based on the correlation coefficient, the observed correlation was defined as weak (0-0.3), moderate (0.3–0.7), or strong (0.7-1.0) [16]. Next, multiple linear regression analysis was conducted to report the prediction of APPE performance (APPE GPA) based on the capstone assessment elements including the drug competency score, calculations competency score, OSCEs, capstone GPA, and PCOA score. The multiple linear regression analysis was conducted using SPSS V.28.0.1.0 (IBM Corp., Armonk, NY) data analysis software.

A cutoff of 73%, which corresponds to grade C, was used for the determination of failure in the competency exams and the OSCEs. This cutoff was complimentary to the capstone course syllabus which also outlined 73% as the threshold to demonstrate competency in most assessments and an overall course grade of 73% was required of students to pass the capstone course. To identify individual predictors of preparedness for APPEs, the GPA earned over APPEs was compared between students failing (< 73%) and passing (≥ 73%) their competency exams during the capstone course using the Mann-Whitney U test. Similarly, the APPE GPA of students was categorized based on the PCOA exam score being higher or lower than the reference group score and compared using the Mann-Whitney U test. Descriptive analysis, the Spearman rank correlation analysis, and the Mann-Whitney U test were conducted using SPSS V.28.0.1.0 (IBM Corp., Armonk, NY). Data was marked significant at *p* ≤ 0.05 for statistical analysis. The research design was reviewed by the University of Findlay’s Institutional Review Board (IRB) and this study was deemed exempt from IRB review (#1666).

## Results

Cumulative data from the two most recent graduating classes was used for this retrospective analysis (*n* = 105). Descriptive analysis of the student data over these two years, as summarized in Table 1, indicates that the majority of the student performance during APPEs, as graded by their preceptors, was outstanding. The overall mean GPA during APPEs for the analyzed dataset was found to be 3.88. For the APPE preceding capstone course, the mean overall course grade was 3.55. As summarized in Table 1, for the cohort data analyzed in this study, the mean student score on the PCOA exam was higher than the corresponding reference group score. Student performance on PCOA, OSCEs, pharmacy calculations competency exam, and top drug competency exam are also summarized in Table 1. While all students passed (> 73%) the OSCEs comfortably (average score ∼ 92%), considerable variation was observed in student performance on the PCOA exam score and the two competency (top drug and pharmacy calculations) examinations of the capstone course.

**Table 1:** Descriptive analysis of student data (class of 2021 and 2022) from this retrospective study

Category	*n*	Mean ± Std. Dev.	Median
APPE– overall GPA (36 credit hours)	105	3.88 ± 0.17	3.96
PCOA score	103	365.7 ± 40.2	363
**Capstone**			
Capstone Course Grade	105	3.55 ± 0.45	3.67
Top Drug Competency Exam (%)	105	86.51 ± 9.19	88.31
Calculations Competency Exam (%)	105	83.35 ± 14.61	85.0
OSCEs– overall score (%)	105	91.75 ± 3.76	91.63

As summarized in Table 2, the Spearman rank correlation analysis revealed a significant (*p* < 0.001) positive correlation (moderate) between the overall capstone course grade and their performance during APPEs (ρ = 0.547). A significant and positive correlation was also observed between student performance on the top drug (ρ = 0.427; *p* < 0.001) and pharmacy calculations (ρ = 0.221; *p* = 0.02) competency exams and their overall GPA earned over the nine APPE rotations. Intuitively, performance on the OSCEs had a significant positive correlation, of moderate intensity, with the APPE GPA as well (ρ = 0.430; *p* < 0.001). Lastly, student performance on the PCOA exam had a moderate positive correlation with student performance during APPEs (ρ = 0.369; *p* < 0.001). Upon comparing the assessed variables, the capstone course grade had the strongest positive correlation with APPE grades followed by student performance on the OSCEs, the top drug competency exam, and the PCOA exam. Amongst the analyzed parameters, student performance on the pharmacy calculations competency exam of the capstone course had the weakest positive correlation with the preceptor-assigned APPE grades.

**Table 2:** Summary of findings from Spearman rank correlation analysis

		Top Drug Competency	Calculations Competency	OSCEs	Capstone GPA	PCOA score
**APPEs - GPA**	Correlation Coefficient (ρ)	0.427	0.221	0.430	0.547	0.369
	*P* value	**< 0.001**	**0.023**	**< 0.001**	**< 0.001**	**< 0.001**
	*N*	105	105	105	105	103

The multiple linear regression model yielded a significant regression equation: (F(5, 97) = 5.62, *p* < 0.001), with an R2 = 0.225 (adjusted R2 = 0.185) suggesting that the regression model was a good fit for the analyzed data. The analyzed independent variable accounted for a 22.5% variance in APPE GPA. As outlined in Table 3, capstone GPA emerged as a significant predictor (β = 0.155; *p* = 0.019) of APPE GPA amongst the tested variables.

**Table 3:** Regression results for a model of predictors of APPE performance (GPA)

Model	Unstandardized Coefficients		Standardized Coefficients	t	Sig.
	B	Std. Error	Beta		
(Constant)	2.984	0.463		6.448	0.000
Drug_Competency	0.001	0.002	0.047	0.419	0.676
Calculation_Competency	1.436E-05	0.001	0.001	0.012	0.990
OSCEs	0.003	0.006	0.059	0.493	0.623
Capstone_GPA	0.155	0.065	0.399	2.390	**0.019**
PCOA_Score	2.805E-05	0.001	0.006	0.053	0.958

Note: the dependent Variable for this multiple linear regression model was APPE_GPA

Subsequently, student performance on the various assessments was used as a measure to categorize the overall grades earned over APPEs. A cutoff of 73% for the top drug competency exam, pharmacy calculations competency exam, and capstone course grade was used to categorize the APPE GPA. For the PCOA score, the reference group score provided in the school’s PCOA report was used as a cutoff for a similar analysis. As indicated in Table 4, students failing the top drug competency exam in their capstone course had significantly lower (*p* = 0.02) APPE GPA compared to students who passed their top drug competency exam. Similarly, students scoring more than the reference group on the PCOA exam earned a significantly higher GPA during their APPEs compared to students who scored less than the reference group on the PCOA exam (*p* = 0.004). Interestingly, no significant difference was observed (*p* = 0.097) in APPE performance between students passing and failing the pharmacy calculations competency examination during their capstone course. These results indicate that student performance on the top drug competency examination and the PCOA exam can serve as potential predictors of APPE preparedness among third-year professional pharmacy students. Importantly, similar conclusions could not be drawn about OSCEs because, while performance on OSCEs had a significant positive correlation with APPE GPA, all student scores for OSCEs were well above the threshold of 73% required to meet the minimum requirements of the capstone course. Hence, categorization of GPA earned during APPE based on performance in OSCEs (< 73% or > 73%) was not feasible.

**Table 4:** Summary of results from Mann Whitney U test comparing APPE GPA categorized as per the listed criteria

	Criteria	*N*	Mean ± Std. Dev.	Mean Rank	Z score	*P* value
**APPE-GPA**	Top Drug Competency ≥ 73	94	3.90 ± 0.17	55.23	-2.328	**0.02**
	Top Drug Competency < 73	11	3.78 ± 0.19	33.91		
	Pharmacy Calculations Competency ≥ 73	87	3.90 ± 0.15	55.11	-1.657	0.097
	Pharmacy Calculations Competency < 73	18	3.81 ± 0.26	42.78		
	PCOA score ≥ Reference score	68	3.92 ± 0.13	57.79	-2.905	**0.004**
	PCOA score < Reference score	35	3.81 ± 0.23	40.74		

## Discussion

The present retrospective analysis indicates that student scores on several assessments included within the capstone course had a positive correlation with the overall APPE grades. Similar to other aspects of the capstone course such as the OSCEs and pharmacy calculations, student knowledge about top drugs, as assessed by the top drug competency exam, had a strong positive correlation with students’ APPE GPA. Importantly, compared to other indicators included in the present data analysis, students who performed poorly in the top drug competency exam of the capstone course and the PCOA exam were more likely to have a lower GPA during their APPEs supporting the notion that the top drug competency exam score and the PCOA exam performance can serve as potential indicators of student preparedness for APPEs.

Multiple studies have examined the factors that may successfully predict APPE readiness in students. Success in major case-based assessments, before students start their APPEs, has been reported to have a strong positive correlation with student performance during APPEs. Such assessments, typically, evaluate the ability of students to review patient information and provide therapeutic recommendations for optimal clinical care [3, 17]. While the utilization of OSCE for assessing student knowledge in pharmacy school was found to be widespread [18], a strong correlation between student scores on OSCE and measures of APPE readiness remains debatable [2, 19]. The results from the present data analysis are in alignment with some studies [17, 20, 21] and demonstrate an overall positive correlation between OSCE scores and practice readiness, as measured by the GPA earned during APPEs. However, given the relatively high scores by almost all students, student performance on OSCEs did not stand out as a reliable measure for predicting student success during APPEs in the present data analysis.

Student performance in the PCOA exam has been found to have a positive correlation with student readiness for APPEs [2, 17] as well as with their performance in the North American Pharmacist Licensure Examination (NAPLEX) [22–24]. While an overall inconsistency concerning the application of, and curriculum adjustments following, PCOA attempts by pharmacy students was reported in a recent analysis [25], a strong correlation between the cumulative academic performance of third-year professional pharmacy students and their PCOA score is well-documented [26, 27]. Importantly, the present analysis underscores the utilization of PCOA exam score as a possible predictor of APPE preparedness with a low PCOA score during the third professional year heightening the probability of students underperforming during their APPEs. However, the recent removal of the mandate for pharmacy schools to require the administration of PCOA exam diminishes the direct applicability of conclusions from the present study. Nevertheless, the present data analysis may assist pharmacy schools to conduct comparable data analysis and provide guidance for their future decision regarding the administration of the PCOA exam. Additionally, ACPE guidelines do promote the inclusion of ‘standardized and comparative assessments’ within their curriculum. Therefore, based on the presented analysis, pharmacy institutions can continue to explore alternate standardized assessments within their capstone course to assess APPE preparedness.

Much like the PCOA score, student performance on the top drug competency exam emerged as a strong indicator of APPE preparedness in the present study. While intuitive, published studies examining a correlation between student knowledge about drug information (adverse effects, therapeutic class, etc.) and their performance during APPEs are currently missing. The current work, therefore, underscores the possibility of utilizing the top drug competency exam or a comparable assessment to gauge student readiness for APPEs.

The Capstone course serves as a checkpoint for a comprehensive assessment of student knowledge/application of their clinical skills and the review of drug information critical for providing the best patient care before the start of APPEs. Instinctively, a capstone course has been incorporated within the pharmacy curriculum at several schools to achieve a variety of student outcomes [14]. In our current curriculum, offered over the spring semester of the third professional year, the capstone course provides an opportunity to assess the pharmacotherapy knowledge and clinical problem-solving skills of pharmacy students. APPE performance, on the other hand, is frequently rated based on the overall grades assigned by preceptors [3, 4, 17]. The results from the present study demonstrate that, amongst the analyzed variables, the overall capstone course grade has the strongest positive correlation with student success during their APPEs. The presented correlation between assessment elements of the capstone course and APPE grades assigned by the preceptor, therefore, provides a measure of the practice readiness of pharmacy students.

The present study has a few limitations. For instance, the data was collected from only 2 cohorts of Pharm.D. graduates. While the collected data represents a promising pilot study, a longitudinal study will help establish the relevancy of the assessed variable on APPE performance. A consistent assessment strategy for the capstone course in the coming years will allow for further data collection thereby, expanding the sample size for the assessed criteria including, for example, the number of students failing the top drug competency exam. Also, the analyzed data comes from an individual school of pharmacy which rules out the possibility to account for the subjectivity of APPE grading by preceptors across the state/several states. Conducting comparable analysis of student data from 2 or more institutions will assist in expanding the range of data points such as the APPE GPA which was averaging closer to 3.9/4.0 in the current analysis.

## Conclusions

In conclusion, the present analysis corroborates previous findings that a standardized assessment (e.g., PCOA) can serve as a potential indicator of student preparedness for and performance during APPEs. In addition, for the first time, the present retrospective study underscores the relevance of the top drug competency exam as an assessment tool to both assess and improve student preparedness for APPEs.

## Data Availability

The dataset analyzed as part of the current study is available from the corresponding author upon reasonable request.
